# Amino Acid Substitution K470R in the Nucleoprotein Increases the Virulence of H5N1 Influenza A Virus in Mammals

**DOI:** 10.3389/fmicb.2017.01308

**Published:** 2017-07-11

**Authors:** Lin Chen, Chengmin Wang, Jing Luo, Meng Li, Huimin Liu, Na Zhao, Jingjing Huang, Xili Zhu, Guoyao Ma, Guohui Yuan, Hongxuan He

**Affiliations:** ^1^National Research Center for Wildlife-Borne Diseases, Institute of Zoology, Chinese Academy of Sciences Beijing, China; ^2^University of the Chinese Academy of Sciences Beijing, China; ^3^State Key Laboratory of Stem Cell and Reproductive Biology, Institute of Zoology, Chinese Academy of Science Beijing, China

**Keywords:** highly pathogenic avian influenza, H5N1, nucleoprotein, mutation, virulence, mammalian cells

## Abstract

H5N1 is a highly pathogenic influenza A virus (IAV) and poses a major threat to the public health. The nucleoprotein (NP) has a multiple functions during the viral life cycle, however, the precise role of NP mutants in viral replication and pathogenicity is not completely understood. Here, we attempted to identify five residues in NP that may contribute to viral replication or pathogenicity. Of these, K227R, K229R, and K470R viruses were successfully rescued by reverse genetic, but the K91R and K198R viruses were not viable. A mini-genome assay demonstrated that the NP mutations K91R and K198R significantly decreased the polymerase activity. Moreover, these two mutations resulted in disrupted cellular localization in mammalian cells. Importantly, mutation at position 470 of NP significantly increased its virulence *in vitro* and *in vivo*. These findings demonstrated that the NP protein plays a major role in influenza virulence and pathogenicity, which adds to the knowledge of IAV virulence determinants and may benefit IAV surveillance.

## Introduction

H5N1, a highly pathogenic avian influenza A (HPAI) virus, is a serious public health threat (El-Shesheny et al., 2017; Ly et al., 2017). Up to 5 April 2017, more than 856 humans were infected the H5N1 virus, with at least 452 deaths^1^. Influenza A viruses (IAVs) are enveloped viruses consisting of a negative-sense RNA genome composed of eight segments (Murphy and Webster, 1996), and encode up to 17 proteins: polymerase basic protein 1(PB1), PB1-F2 (Chen et al., 2001), PB1-N40, polymerase basic protein 2 (PB2), polymerase acidic protein (PA), PA-X (Jagger et al., 2012), PA-N155, PA-N182 (Muramoto et al., 2013), hemagglutinin (HA), nucleoprotein (NP), neuraminidase (NA), nonstructural protein1 (NS1), NS2, NS3 (Selman et al., 2012), matrix protein 1 (M1), M2 and M42 (Wise et al., 2012). The IAV particle contains three major components: the envelope, viral ribonucleoprotein (vRNP) core, and M1. The vRNP core consists of NP and the viral RNA (vRNA) polymerase that includes PB1, PB2, and PA, which together constitute the functional templates for replication and transcription of the viral genome (Honda et al., 1988).

Multiple amino acids residues in proteins of influenza A virus have been identified as the critical determinants for virulence in mammalian hosts. Amino acids 627K and 701D in the PB2 protein increased the polymerase activity and the virulence of the influenza virus in mammalian hosts (Tarendeau et al., 2008; Bussey et al., 2010). PB1 residues 473V and 598P increased the polymerase and virulence in mammalian cells (Xu et al., 2012). PA mutations P103H and S659L reduced viral replication in mammalian cells and attenuated pathogenicity in mice (Zhao et al., 2016). In addition to the polymerase complex, the HA is another major determinant of the host range of influenza A virus (Bottcher-Friebertshauser et al., 2014), and several basic amino acids at the HA cleavage sites contribute to high pathogenicity and induce systematic spread of the viruses in mammals (Chen et al., 1998). Moreover, T at position 215(T215) of the NS1 protein of the H3N2 virus is important for viral replication (Hale et al., 2009). All recombinant viruses containing M1 mutants (Y132A/F/D) were unable to be rescued (Wang et al., 2013). These studies suggested that viral pathogenicity was affected by amino acid mutations in viral proteins.

The NP is highly conserved among influenza A viruses. This protein is a major structural component of the viral particle and encapsulates the viral RNA (Portela and Digard, 2002). It contains a RNA-binding region at its N terminus and two domains, which are responsible for NP-NP self-interaction, at residues 189 to 358 and 371 to 465 (Ng et al., 2009). NP is a shuttle protein between the nucleus and cytoplasm (Neumann et al., 1997). In the early phases of infection, NP accumulates in the nucleus. During viral assembly and maturation, NP is exclusively distributed in cytoplasm. Several studies have reported that NP protein is associated with the virulence in mammalian cells. To assess the conserved amino acids, (Li et al., 2009) used a comprehensive analysis to identify 15 amino acid residues that are crucial for viral-genome replication, and 17 amino acid substitutions had no or little effect on viral growth *in vitro*. Several post-translational modifications affected the NP functions. K184 is an ubiquitination site on NP and this residue was crucial for virus RNA replication (Liao et al., 2010). K4 and K7 of the NP protein are the SUMO acceptor sites, and the mutant WSN-NP K4,7R virus was highly attenuated and had aberrant nuclear/cytoplasmic trafficking dynamics (Han et al., 2014). Phosphorylation of the NP protein was first observed many years ago (Kistner et al., 1989). It was reported that S9, Y10, S165, and Y296 were the phosphorylation sites. S9 and Y10 controlled nuclear import of NP, and mutation of these two sites dramatically decreased viral titers in cell culture and modulation of the viral polymerase activity in a mouse model (Zheng et al., 2015). Although many efforts have already been made to understand the role of NP residues, the precise role of some NP mutants in viral replication and pathogenicity remains unclear.

In our previous studies, five residues (lysine 91, 198, 227, 229, and 470) of NP were shown to be modified by acetylation using mass spectrometry (unpublished data). In the current study, we focused on the five residues of NP in viral replication and pathogenicity. Using reverse genetics, we successfully rescued K227R, K229R, and K470R mutant viruses and analyze the virulence of the recombinant viruses *in vitro* and *in vivo*. For the K91R and K198R mutations that did not support viral growth, we studied the cellular distribution in mammalian cells.

## Materials and Methods

### Cells and Viruses

MDCK cells, 293T cells and A549 cells were cultured in Dulbecco’s minimal essential medium (DMEM) supplemented with 10% fetal bovine serum (FBS), 100 IU/ml penicillin and 100 μg/ml streptomycin. The A/Qing Hai/environment/2005 (H5N1) strain was propagated in 10-day-old embryonated chicken eggs.

### Biosafety and Ethics Statement

All experiments involving H5N1 viruses were performed in a Biosafety Level 3(BSL-3) containment laboratory in the Research Center for Wildlife Diseases, which was approved by the Chinese Academy of Science. All animal experiments were performed in compliance with the Guide for the Care and Use of Laboratory Animals of the Ministry of Science and Technology of the People’s Republic of China. The protocol was approved by the Committee on the Ethics of Animal Experiments of the Institute of Zoology, Chinese Academy of Sciences (approval number: IOZ-15042). Mice were euthanized with CO_2_ to minimize animal suffering if the weight loss of more than 20% of the body weight was observed.

### Plasmids Construction

The full-length NP (A/Qing Hai/environment/2005(H5N1)) was cloned into the pcDNA3.1-Myc and pHW2000 vectors. Lysine (K) to arginine (R) mutations in the NP genes were introduced using the QuikChange site-directed mutagenesis kit (Stratagene) with primers designed following the manufacturer’s protocol. All primers used in this study are shown in **Table 1**. All plasmid constructs were sequenced to confirm the sequence integrity.

**Table 1:** Primers used for site-directed mutagenesis.

Primer name	Sequence (5′–3′)
NP-K91R-F	GGACCCAAAGAGAACTGGAGGTCCAATCTACC
NP-K91R-R	GGTAGATTGGACCTCCAGTTCTCTTTGGGTCC
NP-K198R-F	TCGGATGATAAGACGAGGGATCAATGATCG
NP-K198R-R	CGATCATTGATCCCTCGTCTTATCATCCGA
NP-K227R-F	CAACATCCTCAGAGGGAAATTCCAAACAGCAG
NP-K227R-R	CTGCTGTTTGGAATTTCCCTCTGAGGATGTTG
NP-K229R-F	CCTCAAAGGGAGATTCCAAACAGCAGCACA
NP-K229R-R	TGTGCTGCTGTTTGGAATCTCCCTTTGAGG
NP-K470R-F	CTCTCGGACGAAAGGGCAACGAACCCGATC
NP-K470R-R	GATCGGGTTCGTTGCCCTTTCGTCCGAGAG


### Generation of Recombinant Influenza Viruses by Reverse Genetics

Recombinant viruses were rescued by eight-plasmid reverse genetics followed by two-round plaque purification and propagation on MDCK cells as previously described (Hoffmann et al., 2000). Briefly, the NP gene from A/environment/Qinghai/1/2008 (H5N1) (as wild type) or the NP gene with the mutation K91R, K198R, K227R, K229R, or K470R was co-transfected with the plasmids encoding the other seven genes from the A/environment/Qinghai/1/2008 (H5N1) virus into 293T cells and MDCK cells. The medium was replaced 24 h later with DMEM plus 1 μg/ml tosylsulfonyl phenylalanyl chloromethyl ketone (TPCK)-treated trypsin. At 72 h after transfection, the supernatant containing the recombinant viruses was harvested and then centrifuged at 2,000 × *g* for 10 min to remove the cell debris.

### Plaque Assay

Monolayer cultures of MDCK cells in 6-well plates were washed twice with phosphate-buffered saline (PBS). The cells were infected with serial 10-fold dilutions of virus for 1 h 37°C and shaken at every 15 min. The virus inoculums were then overlaid with agar overlay medium (DMEM supplemented with 0.6% low-melting-point agarose and 1 μg/ml TPCK-treated trypsin) and incubated at 37°C. After 3 days, the cells were fixed with 10% formaldehyde and stained with 0.5% crystal violet solution.

### Viral Replication Kinetics in Mammalian Cells

A549 cells were inoculated with the indicated virus at a multiplicity of infection (MOI) of 1/0.1 plaque-forming units per cell. After 1 h of incubation at 37°C, cells were washed twice with 1× PBS and were maintained in fresh medium for virus replication. The supernatants were collected at 3, 6, 9, 12, 24, 36, and 48 h after virus infection. Virus titers in the supernatants were detected by TCID_50_.

### Mini-genome Assay

The 293T cells in 6-well plates were transfected with plasmid (PB2/pHW2000, PB1/ pHW2000, PA/ pHW2000, and NP/pHW2000 or its mutants) DNA (1 μg each) and a luciferase RNA expression (vNS-luc/pHH21) vector and pRL-TK(50 ng), which expresses *Renilla* luciferase as an internal control. The cells were lysed at 36 h post-transfection, and luciferase activity was measured using the Dual Luciferase Assay System (Promega) according to the manufacturer’s protocol. Polymerase activity is calculated by normalizing the firefly luciferase activity to the *Renilla* luciferase activity. Polymerase activity of wild-type was set to 100%.

### Confocal Microscopy

First, 293T cells were seeded on coverslips and grown to 80–90% confluence. Cells were then cotransfected with 1 μg of NP WT or mutants plasmids. At 9, 24, and 30 h post-transfection, the coverslips carrying cells were washed with PBS and then fixed with 4% paraformaldehyde and permeabilized with 0.1% Triton X-100. Cells were then blocked with 1% bovine serum albumin (BSA) and stained with NP antibodies for 2 h at RT, washed 3 times with PBS, and incubated with an Alexa Fluor 488-conjugated goat secondary antibody against rabbit IgG (1:500 in PBS) for 1 h. The cells were then incubated with PBS containing DAPI (4, 6-diamidino-2-phenylindole) for 5 min and then washed three times with PBS. The excitation and emission wavelengths were set at 488 nm and 519 nm, respectively. Prepared samples were observed under a confocal laser scanning microscope (Leica 780).

### RNA Isolation, Reverse Transcription, and Viral mRNA, cRNA and vRNA Quantification

Total RNA from infected cells was extracted using TRIzol (Invitrogen) according to the manufacturer’s instructions. The cDNAs of the corresponding virus mRNA, cRNA and vRNA were first reverse-transcribed by using the GoScript Reverse Transcription System (Promega) with the tagged primers shown in **Table 2**. The analysis of relative vRNA, cRNA, and mRNA expression was performed using an ABI 7500 machine. The cycling conditions comprised an initial denaturation step of 3 min at 95°C, followed by 40 two-step cycles (95°C for 5 s and 60°C for 60 s). Dissociation curve analysis was performed after each assay to ensure specific target detection. The cellular β-actin mRNA in the infected cells was used as an internal control.

**Table 2:** Primers used for reverse transcription and Real-time PCR.

Target	Purpose	Primer name	Sequence (5′–3′)
vRNA	Reverse	vRNAtag-NP	GGCCGTCATGGTGGCGAAT
	transcription		AAGGACCAGGAGTGGAGGAAA
	Real-time PCR	vRNAtag	GGCCGTCATGGTGGCGAAT
		vRNA-NP-1315R	CAGATGTTCTGCCCTCCGTA
cRNA	Reverse	cRNAtag-NP	GCTAGCTTCAGCTAGGCATC
	transcription		AGTAGAAACAAGGGTATTTTTCT
	Real-time PCR	cRNAtag	GCTAGCTTCAGCTAGGCATC
		cRNA-NP-1344F	GGAAAGTGCCAAACCAGAAGA
mRNA	Reverse	mRNAtag-NP	CCAGATCGTTCGAGTCGT
	transcription		TTTTTTTTTTTTTTTT
	Real-time PCR	mRNAtag	CCAGATCGTTCGAGTCGT
		mRNA-NP-1344F	GGAAAGTGCCAAACCAGAAGA


### Western Blot

Cells were washed once with PBS and lysed in lysis buffer (50 mM Tris-HCl pH 7.6, 150 mM NaCl, 0.1% SDS, 1% Triton-100) further supplemented with protease inhibitor (Roche). The whole cell lysates were resolved by SDS-PAGE. Proteins were transferred to nitrocellulose membrane and blocked with 5% nonfat milk in TBST. The membrane was probed with primary antibody followed by secondary antibody at room temperature for 2–4 h. The membrane was washed at least three times with TBST (0.1% Tween 20) after incubation with each antibody and developed with the Dura chemiluminescent kit (Millipore).

### Animals

Female 4- to 6-week-old BALB/c mice (Stanford, China) were lightly anesthetized with CO_2_ and inoculated intranasally with a 50 μl volume of the indicated dose. The mice in the control group were inoculated with PBS. Mice were euthanized on day 3 post inoculation, and lungs were collected and titrated for viral titer in MDCK cells and hematoxylin and eosin (HE) staining. The remaining mice (*n* = 4) were monitored daily for 14 days and assessed for body weight and mortality.

### Statistical Analysis

Data are expressed as the mean ± SDs. Student’s *t*-test was used for statistical comparisons; *p* < 0.05 was considered significant.

## Results

### Generation of NP K227R, K229R, and K470R Mutant Viruses

**FIGURE 1:**
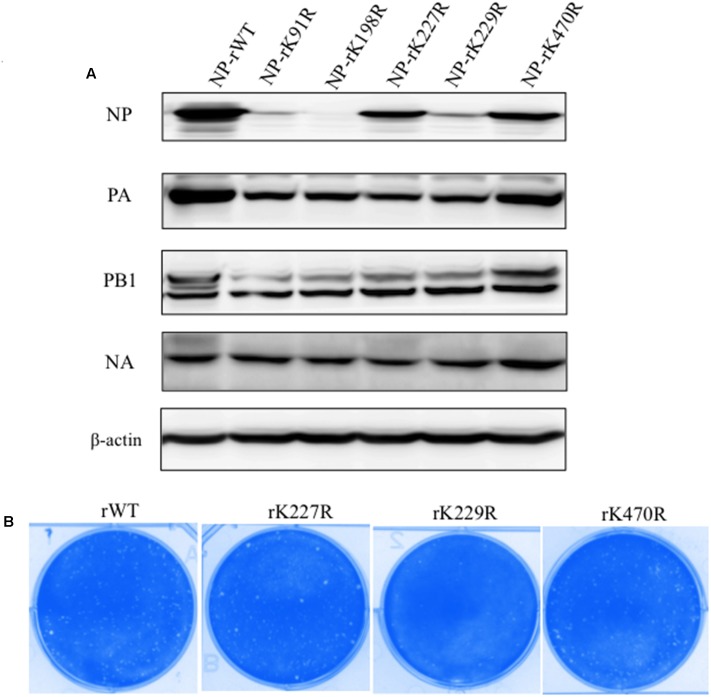
Rescue mutant viruses by reverse genetics. **(A)** MDCK and 293T cells were cotransfected with eight plasmids of the H5N1. At 72 h p.t., the transfected cells were lysed for western blot analysis. PA, NP, PB1, and NA were detected with respective antibodies. **(B)** The plaque morphology of NP-rWT, NP-rK227R, NP-rK229R, and NP-rK470R viruses.

To determine the function of five lysines (K91, K198, K227, K229, and K470) in NP, we performed site-directed mutagenesis to replace the lysine (K) residues with arginine (R) and generated the K91R, K198R, K227R, K229R, and K470R recombinant viruses using plasmid-driven reverse genetics. MDCK and 293T cells were co-transfected with NP mutants and the other seven plasmids, and at 72 h post transfection, viral proteins within transfected cells were detected by western blot. The expression of viral protein (PB1, NA and PA) was almost similar in all of the wild-type (WT) and mutant NP-transfected cells, although the NP expression level in K91R, K198R and K227R was lower than that of wild-type (**Figure 1A**). The NP rWT, rK227R, rK229R, and rK470R viruses were successfully rescued. The plaque sizes of the rK227R, rK229R and rK470R mutant viruses were similar to that of the rWT virus (**Figure 1B**). The number of plaque in rK470R was higher than those of rWT. In contrast, the K91R and K198R mutant viruses were not viable in three independent experiments, suggesting the two mutations in NP were critical for the viral life cycle.

### Effect of NP Mutants on the Polymerase Activity

**FIGURE 2:**
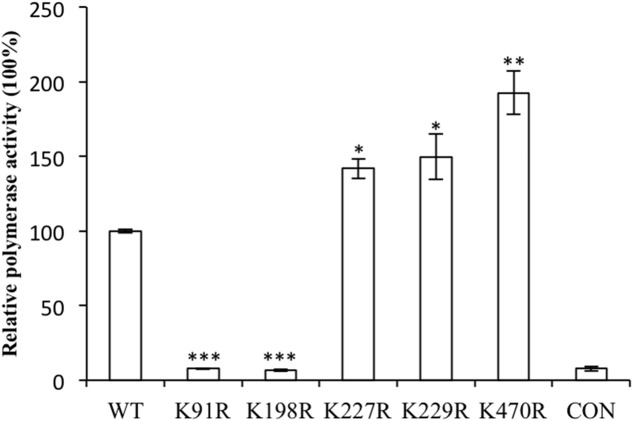
Nucleoprotein (NP) mutants affected the influenza A virus genome RNA replication using the mini-replicon reporter system. HEK293T cells were transfected with pPolI–Luc and plasmids for the expression of the viral PB2, PB1, PA and NP (wild-type or mutants). Renilla luciferase was used as an internal control. Luciferase assays were performed at 24 h after transfection. The data were normalized relative to the values detected in the cells transfected with wild-type NP. Values are the mean ± SEM of three separate experiments. ^∗^*P* < 0.05; ^∗∗^*P* < 0.01; ^∗∗∗^*P* < 0.001.

The polymerase complex of influenza A virus, composed of PA, PB1, PB2, and NP, is essential for viral transcription and replication (Honda et al., 1988). Minireplicon assays were performed to evaluate the impact of the NP substitutions K91R, K198R, K227R, K229R, and K470R on the polymerase activity in human embryonic kidney (293T) cells at 37°C. As shown in **Figure 2**, the K91R and K198R mutants displayed substantially lower luciferase activity than that of WT, while the K227R, K229R, and K470R mutants increased the polymerase activity by 1.4-, 1.5-, and 1.9-fold, respectively. Our results indicated that K91R and K198R mutants likely lost the ability to support viral polymerase activity, resulting in the growth defects of the mutant viruses.

### K91 and K198 Disrupted the Cellular Distribution of NP

**FIGURE 3:**
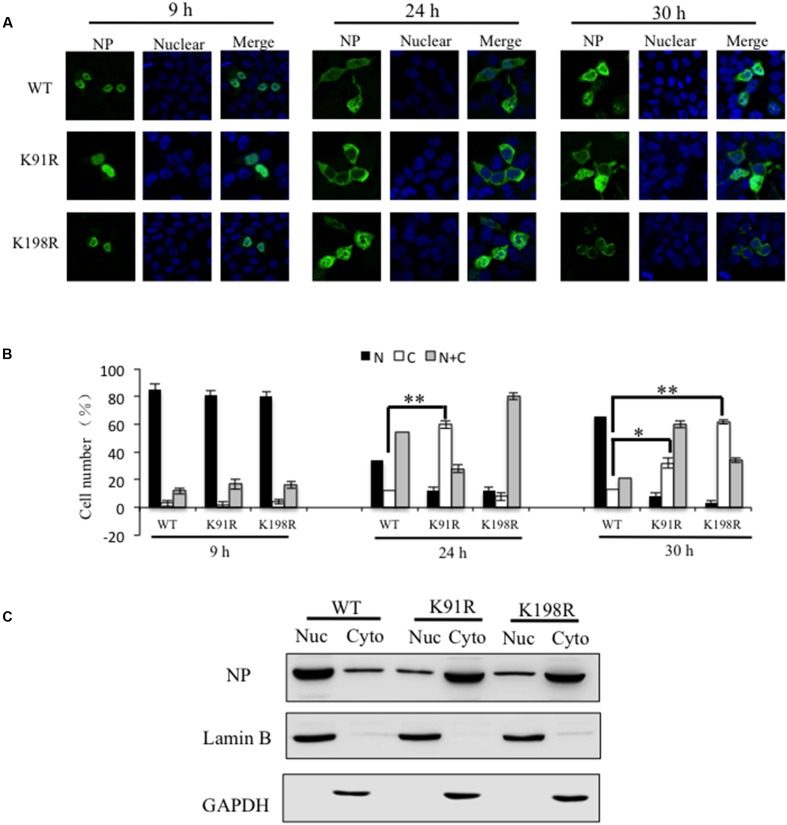
K91R and K198R mutants disrupted the cell distribution of NP. **(A)** The localization of WT NP and its mutants (K91R/K198R) was determined using IFAs. The 293T cells were transfected with plasmids expressing mutant NPs or WT NP. At 9, 24, and 30 h p.t., the transfected cells were fixed and stained with anti-NP antibody (green). The nucleus was stained with DAPI (blue). Scale bar: 10 μm. **(B)** At least 200 cells in each group for three independent assays were scored as predominantly nuclear (N), nuclear and cytoplasmic (N+C), or predominantly cytoplasmic (C). *P-*values were determined using Student’s *t*-test by comparing to the rWT virus group. ^∗^*P* < 0.05; ^∗∗^*P* < 0.01. **(C)** 293T cells were transfected with a plasmid expressing WT NP, K91R or K198R mutant and harvested at 30 h p.t. The cells were separated into the nuclear fraction (Nuc) and the cytoplasmic fraction (Cyto). Each fraction was examined by Western blotting.

Nucleoprotein is a shuttle protein between the nucleus and the cytoplasm. Mutations of several residues, such as the R8A and Y296F mutations, cause cytoplasmic accumulation of NP (Zheng et al., 2015). We hypothesized that K91 and K198 would regulate the cellular distribution of NP. To test our hypothesis, imumunofluorescence assays (IFAs) were carried out to examine the cellular localization of NPs. Plasmids encoding NP WT, K91R or K198R were transfected into 293T cells, and the transfected cells were fixed at 9, 24, and 30 h p.t. Imaging revealed that NP WT exhibited nuclear localization at 9 and 24 h p.t. (**Figures 3A,B**). However, K91R and K198R mutants displayed cytoplasmic localization at 24 and 30 h p.t., respectively (**Figures 3A,B**). In addition, the results of cytoplasmic and nuclear fractionation experiments also demonstrated that K91R and K198R mutations increased the cytoplasmic distribution of NP (**Figure 3C**). We speculated that disrupting NP cellular distribution was associated with the lower RNP activity. Together, our results showed that NP-K91R/K198R disrupted the cellular location of NP.

### Effects of K227R, K229R, and K470R on Virus Growth *In Vitro*

**FIGURE 4:**
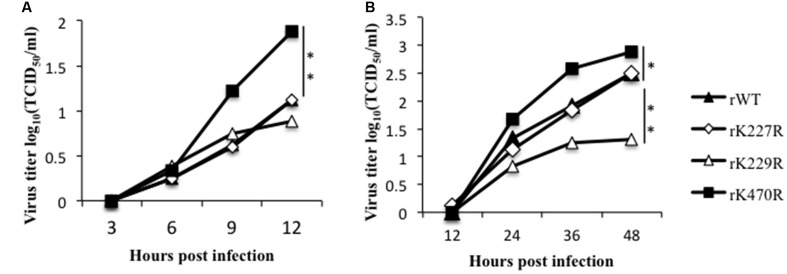
The characteristics of the K227R, K229R and K470R mutant recombinant viruses *in vitro*. **(A)** A549 cells were infected with rK227R, rK229R, rK470R or rWT virus at an MOI of 1. The supernatants were collected at 3, 6, 9 and 12 h after infection and then titrated in MDCK cells by TCID_50_. **(B)** A549 cells were infected with rK227R, rK229R, rK470R or rWT virus at an MOI of 0.1. The supernatants were collected at 12, 24, 36 and 48 h after infection, and then titrated in MDCK cells by TCID_50_. Values are the mean ± SEM of three separate experiments. *P*-values were determined using Student’s *t*-test by comparing to the rWT virus group. ^∗^*P* < 0.05; ^∗∗^*P* < 0.01.

To evaluate the impact of the substitutions on virus growth, we inoculated recombinant viruses rK227R, rK229R, and rK470R into A549 cells at an MOI of 1 and harvested supernatants at 3, 6, 9, and 12 h post-infection (hpi). As shown in **Figure 4A**, viral titers of rK470R were 100-fold and 50-fold higher than those of rWT at 9 and 12 hpi, respectively. Viral titers of rK229R were lower than that of rWT at 12 hpi, while those of rK227R were similar with the rWT. Therefore, the K470R mutants enhanced virus growth of H5N1 in mammalian cells in a single cycle growth curve. For multiple cycle growth curves, A549 cells were inoculated at an MOI = 0.1, and the supernatants were harvested at 12, 24, 36, and 48 hpi. The rK227R and rWT viruses had the similar growth kinetics in A549 cells. In contrast, the titer of rK470R was significantly increased compared to that of the rWT virus at 36 and 48 hpi (20-fold and 16-fold, respectively), and the rK229R virus exhibited significantly reduced replication compared to the rWT virus (**Figure 4B**). Together, our results indicated that the K470R mutant increased the virus growth in mammalian cells, while the K229R suppressed the virus growth and the K227R had no effect.

### Effects of K227R, K229R, and K470R on the Synthesis of Viral mRNA, cRNA, and vRNA

**FIGURE 5:**
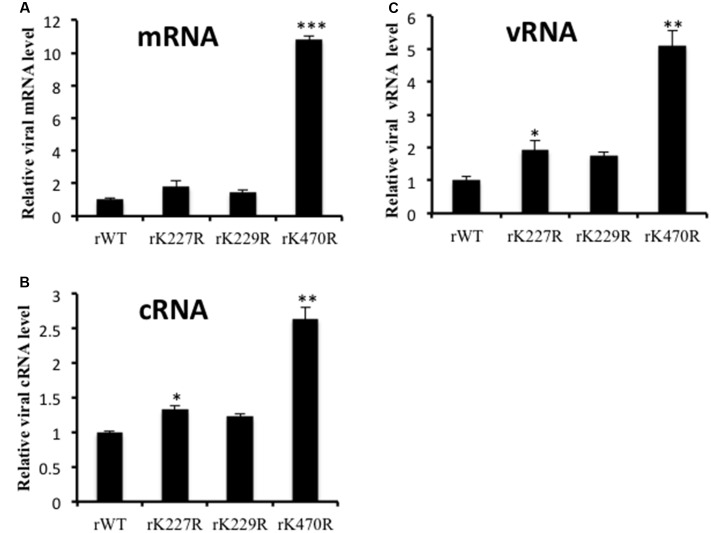
Quantification of viral mRNA, cRNA and vRNA. A549 cells were infected with rK227R, rK229R, rK470R, or rWT virus at an MOI of 1. At 6 h after infection, total cellular RNA was analyzed by two-step real-time RT-qPCR to discriminate and quantify viral NP mRNA **(A)**, cRNA **(B)** and vRNA **(C)**. The reactions were carried out in triplicate and normalized to the levels of cellular β-actin mRNA. The value for rWT was set to 1. Data represent the mean ± SD values. ^∗^*P* < 0.05; ^∗∗^*P* < 0.01; ^∗∗∗^*P* < 0.001, compared with that of rWT by Student’s *t*-test.

In influenza virus-infected cells, the vRNPs direct two types of RNA synthesis: mRNA synthesis (transcription) and vRNA amplification (replication). The vRNA template is copied to form full-length positive-stranded RNA (cRNA), which forms the template for synthesis of vRNA segments for amplification of mRNA synthesis and packaging into progeny virions (York and Fodor, 2013). We investigated whether these substitutions would affect viral vRNA, cRNA, and mRNA synthesis. A549 cells were infected with the rWT, rK227R, rK229R or rK470R virus at an MOI of 1 for 6 h, and the synthesis of viral NP mRNA, cRNA, and vRNA was quantified. The results showed that the rK227R, rK229R, or rK470R viruses produced higher levels of mRNA (**Figure 5A**), cRNA (**Figure 5B**), and vRNA (**Figure 5C**) than those of rWT. Among these substitutions, K470R produced the highest levels of mRNA, cRNA, and vRNA, which were 10.8-fold, 2.6-fold, and 5.1-fold greater than those of rWT, respectively (**Figure 5**). Taken together, these results indicated that K470R enhanced viral transcription and replication by increasing the synthesis of viral mRNA, cRNA, and vRNA in human cells.

### Effects of K227R, K229R, and K470R on Viral Pathogenicity in Mice

**FIGURE 6:**
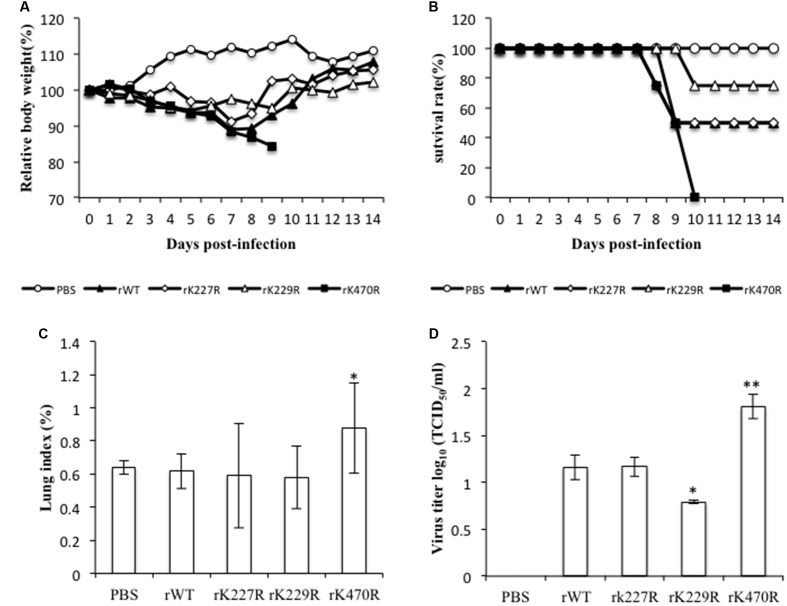
The characteristics of the K227R, K229R, and K470R mutant recombinant viruses *in vivo*. **(A,B)** BALB/c mice (eight/group) were inoculated intranasally with rWT, rK227R, rK229R, and rK470R viruses at a dose of 1 × 10^4^ TCID_50_. Infected animals were monitored daily for body weight changes **(A)** and survival **(B)**. **(C,D)** Three mice from each group were euthanized on day 3 postinfection to determine the lung index **(C)** and lung viral titer **(D)**. The lung index was calculated as the lung wet weight/body weight × 100. Values are the mean ± SEM. of three separate experiments. *P*-values were determined using Student’s *t*-test by comparing to the rWT virus group. ^∗^*P* < 0.05; ^∗∗^*P* < 0.01.

To assess whether the phenotypes of mutant viruses *in vitro* manifested *in vivo*, we evaluated the growth and pathogenicity of these viruses in a mouse model. Seven BALB/c mice each were intranasally inoculated with 1 × 10^4^ TCID_50_ of rWT or mutant rK227R, rK229R, or rK470R virus. Control mice were inoculated with PBS. Three mice from each group were euthanized on day 3 post-infection, and the remaining four mice were monitored daily for 14 days for changes in body weight and for death. Mice infected with the rWT virus rapidly lost weight, and two of four infected mice died by day 8. The weight change pattern and the survival rate of rK227R were similar to that of rWT-infected mice. However, only one of four mice infected with rK229R died by day 10. In contrast, four rK470R virus-infected mice died by day 8 (**Figures 6A,B**), consistent with the increased the virus growth of the K470R in mammalian cells. We next determined lung index and viral titers on day 3 post-infection. The lung index and the virus titer of rK470R-infected mice were higher than other groups (**Figures 6C,D**).

**FIGURE 7:**
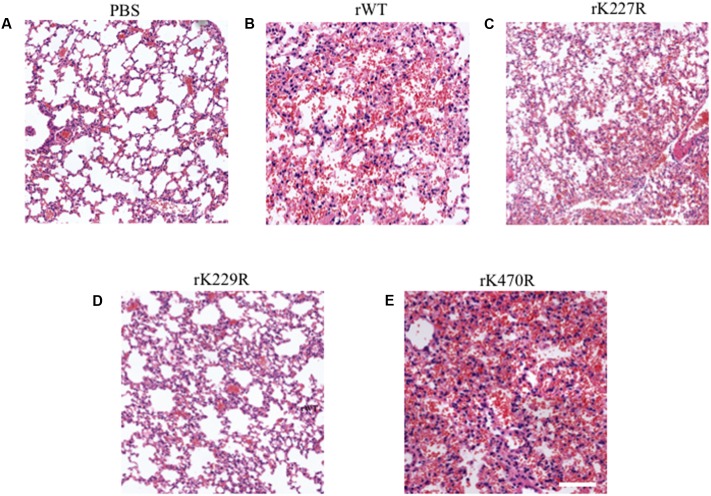
Histopathologic changes in the lungs of infected mice. The images shown are hematoxylin- and eosin-stained sections of lungs from BALB/c mice infected with PBS **(A)**, rWT **(B)**, rK227R **(C)**, rK229R **(D)**, or rK470R **(E)** virus. Original magnification, ×20.

To further compare the pathogenicity of these viruses *in vivo*, we performed histological analysis of lungs from mice infected with the rWT, rK227R, rK229R, or rK470R virus on day 3 post-infection (**Figure 7**). Control group mice displayed thin alveolar walls and large air spaces, which indicated normal lung function (**Figure 7A**). Mice infected with rWT showed inflammation with immune infiltrates in the alveolar interstitial spaces and mild hemorrhage (**Figure 7B**). Similarly, infection with rK227R caused mild hemorrhage (**Figure 7C**). In contrast to the rWT, infection with rK229R exhibited mild inflammation (**Figure 7D**). In addition, infection with rK470R led to a significant increase of cellular infiltration and severe hemorrhage (**Figure 7E**). Collectively, these findings demonstrated the enhanced effect of rK470R on the pathogenicity of subtype H5N1 avian influenza in mice.

## Discussion

Potential mutations in influenza A virus that increase pathogenicity have not been thoroughly examined. In this study, we found that several lysine-to-arginine substitutions of the NP protein markedly affected viral virulence *in vitro* and *in vivo*. Compared with the rWT virus, the rK470R mutant virus showed enhanced virulence. The crystal structure of NP of H5N1 has been determined (Ng et al., 2008). The amino acid at position 470 was exposed on the surface of the trimeric NP, indicating the K470 residue had a critical role. The replacement of lysine with arginine could affect the interaction of NP with other viral proteins. Therefore, K470R enhanced the polymerase activity in minireplicon assays, and the vRNA, cRNA, and mRNA levels were higher than those of WT. By sequence alignment and analyses, we showed that the amino acid at position 470 of NP is polymorphic among influenza A viruses, but the 91, 198, 227, and 229 residues were conserved (**Table 3**). Our inspection of 13773 influenza A virus NP sequences (obtained from the Influenza Research Database^2^) revealed that most NP proteins encoded NP-470K. Interestingly, 92.6% of human H1N1 isolated before March 2009 presented NP-470R, whereas 99.7% of avian-origin H1N1 viruses harbored NP-470K (**Table 4**). In March 2009, a novel influenza A virus [influenza A (H1N1) 2009] emerged in Mexico and rapidly spread worldwide. The virus is more pathogenic than seasonal influenza viruses and caused more than 18,000 deaths (Scalera and Mossad, 2009; Stech et al., 2010). During the pandemic, K was substituted with R. We speculated that the NP-470 R to K substitution in influenza A (H1N1) 2009 may be one of the contributing factors that account for the higher pathogenicity and mammalian adaptation. The roles of NP-K470R in influenza H1N1 2009 in viral life cycle, pathogenicity and mammal transmissibility will be examined in our further work. In this study, we revealed that K470R substitution in H5N1 facilitated viral replication and pathogenicity *in vitro* and *in vivo*. Our findings may be helpful for the surveillance of IAVs.

**Table 3:** Conservation of NP residues 91, 198, 227, 229, and 470 in influenza A viruses.

	Position of amino acid in NP				
	
Residues	91	198	227	229	470
K	99.9%	99.9%	99.9%	100%	86.4%
R	0.1%	0.1%	0.1%	0	13.7%


**Table 4:** NP residues 470 in avian and human influenza A viruses.

	H5N1		H1N1			H7N9	
			
Residues	Avian	Human	Avian	Human		Avian	Human
				Then-2009/03	2009/04-Now		
470K	2712	227	493	113	8127	98	494
470R	0	0	1	1421	87	0	0


The influenza virus RNA polymerase is an important virulence determinant (Watanabe et al., 2013). In the current study, the RNP activity of K227R and K229R was higher compared with WT, but the mutants did not show increased the virus replication or virulence in mammals. These results suggested that in addition to RNP activity, other factors were also associated with the pathogenicity of influenza A, such as viral entry, trafficking to the cell surface, viral assembly and host immune response (Neumann and Kawaoka, 2006; Keleta et al., 2008; Shapira et al., 2009; Bottcher-Friebertshauser et al., 2014). Recent studies demonstrated that G158 mutation of the HA protein enhanced viral productivity and induced stronger host immune, and thereby contributed to the high pathogenicity of H5N1 virus in mice (Zhao et al., 2017). Mutations in the M1 protein also contributed to the virulence of H5N1 influenza viruses in mice (Fan et al., 2009). Though there are many important data in this area, the mechanism of the virulent determinants of influenza A viruses needs to be further explored.

Nucleoprotein can shutter between the nucleus and the cytoplasm because of its nuclear localization signals (NLSs) and nuclear export signals (NESs). Three NESs have been found in NP, NES1 (E24IRASVGKMIDGIGRFYIQMCTELKL49) and NES2 (V183KGVGTMVMELIRMI197), which do not rely on CRM1 for export, and NES3 (P248GNAEFEDLIFL ARSALILRGSVAHKS274), which does (Yu et al., 2012). The three NESs are closely linked to nuclear export of vRNP, and mutation of the NP NESs inhibits the replication of the virus. In addition, there are three NLSs in NP. An unconventional NLS (M1ASQGTKRSYEQM13) is at the N-terminus of the NP. The second NLS (K198RGINDRNFWRGFNGRRTR216) is located in the central region of NP. The third NLS is located between amino acids 320 and 400 (Neumann et al., 1997). The K198R mutation, located in the second NLS, affected the NP nuclear location; polymerase activity was lower, and the recombinant virus K198R was not viable.

K91 is located in the body domain of NP. NP was shown to interact with PB2 through residues 1-161 and 255-465. Viruses with single point mutations at K91R could not be rescued, indicating that the residue is essential for the survival of the virus, probably because of its important roles in PB2 binding.

NP, a major component of vRNP, plays a role in RNA synthesis in the nucleus. Although PB1, PB2 and PA proteins are sufficient to synthesize short RNAs, NP is necessary for synthesis of longer RNA (Honda et al., 1988). NP is known to interact with several viral proteins, such as PB2, PB1, NS1, M1 and NP itself. NP is also believed to be a key adaptor for virus and host-cell interaction. Many efforts have been made to identify host proteins associated with NP (Generous et al., 2014; Sun et al., 2015). The host protein cluster (CLU) was identified as a novel interacting partner with influenza A viral NP during virus infection. Overexpression of CLU attenuated IAV replication in mammalian cells (Tripathi et al., 2013). NP interacted with F actin and caused cytoplasmic retention of NP late in infection (Avalos et al., 1997). The TREX complex adaptor protein Aly/REF interacted with NP, and siRNA knockdown of Aly/REF had little effect on export of NP and no significant reduction in viral titer (Balasubramaniam et al., 2013). Using Gal4-based yeast two-hybrid(Y2H) assay, phosphoribo-sylaminoimidazole succinocarboxamide synthetase (PAICS) was shown to interact with NP. PAICS was found to be significantly up-regulated during an IAV infection (Generous et al., 2014). The study of the interaction between host and NP can provide new ideas for the development of clinical drug target and the prevention of the disease (Kao et al., 2010).

Because influenza viruses have a segmented RNA genome, exchange of segments between different viruses and mutation can occur when different influenza viruses infect the same cell (Wu et al., 2013; Gerber et al., 2014). In our previous studies, we showed that diverse wild bird H9N9 influenza virus genes elevated the virulence of a H7N9 virus (Su et al., 2015). Recently, cases of human infections with newly reasserted influenza A (H7N9) virus have been continuously reported in China (Sha et al., 2016; Shen and Lu, 2017). The emergence and transmission of novel avian IAVs to humans are complicated, which can be due to a series of events, including the reassortment of the virus genome and accumulation of adaptive mutations.

In this study, we analyzed the effect of NP mutants on the virulence of the H5N1 in mammals. Of these, K91R and K198R disrupted the NP cellular location and decreased the polymerase activity. Compared with the rWT, the rK229R mutant exhibited an attenuated virulence, while the rK227R showed the same virulence as that of the rWT. Importantly, the K470R mutant enhanced viral replication and pathogenicity in mammals. The identification of these substitutions may contribute to our understanding of the biology of IAVs. In addition, it may also facilitate the prediction and controlling of future IAV epidemics and pandemics.

## Author Contributions

HH and LC designed this experiment, and wrote this manuscript; LC, JL, ML, JH, XZ, GY, and GM performed the experiments; LC, CW, HL and NZ participated in the data analysis; CW and ML provided some useful suggestion on the project and manuscript revising.

## Conflict of Interest Statement

The authors declare that the research was conducted in the absence of any commercial or financial relationships that could be construed as a potential conflict of interest.

## Footnotes

1 http://www.who.int

2 http://platform.gisaid.org
